# An assessment of the use of patient reported outcome measurements (PROMs) in cancers of the pelvic abdominal cavity: identifying oncologic benefit and an evidence-practice gap in routine clinical practice

**DOI:** 10.1186/s12955-020-01648-x

**Published:** 2021-01-15

**Authors:** Miss Charlotte L. Moss, Ajay Aggarwal, Asad Qureshi, Benjamin Taylor, Teresa Guerrero-Urbano, Mieke Van Hemelrijck

**Affiliations:** 1 King’s College London, School of Cancer and Pharmaceutical Sciences, Translational Oncology and Urology Research (TOUR), Guy’s Hospital, 3rd Floor Bermondsey Wing, Great Maze Pond, London, SE1 9RT UK; 2 Comprehensive Cancer Centre, Guy’s and St Thomas’ NHS Foundation Trust, London, UK; 3 Department of Health Services Research and Policy, London School of Hygiene and Tropical Medicine, London, UK

**Keywords:** Patient reported outcome measurements, Health related quality of life, Prognostic factors, Overall survival, Pelvic abdominal cancers

## Abstract

**Background:**

Patient reported outcome measurements (PROMs) are emerging as an important component of patient management in the cancer setting, providing broad perspectives on patients’ quality of life and experience. The use of PROMs is, however, generally limited to the context of randomised control trials, as healthcare services are challenged to sustain high quality of care whilst facing increasing demand and financial shortfalls. We performed a systematic review of the literature to identify any oncological benefit of using PROMs and investigate the wider impact on patient experience, in cancers of the pelvic abdominal cavity specifically.

**Methods:**

A systematic review of the literature was conducted using MEDLINE (Pubmed) and Ovid Gateway (Embase and Ovid) until April 2020. Studies investigating the oncological outcomes of PROMs were deemed suitable for inclusion.

**Results:**

A total of 21 studies were included from 2167 screened articles. Various domains of quality of life (QoL) were identified as potential prognosticators for oncologic outcomes in cancers of the pelvic abdominal cavity, independent of other clinicopathological features of disease: 3 studies identified global QoL as a prognostic factor, 6 studies identified physical and role functioning, and 2 studies highlighted fatigue. In addition to improved outcomes, a number of included studies also reported that the use of PROMs enhanced both patient-clinician communication and patient satisfaction with care in the clinical setting.

**Conclusions:**

This review highlights the necessity of routine collection of PROMs within the pelvic abdominal cancer setting to improve patient quality of life and outcomes.

## Introduction

The incidence of cancers of the pelvic abdominal cavity (broadly urological, gynaecological, colorectal, gastric, hepatic and pancreatic tumour types) is increasing as population life-expectancy increases [1]. As of 2018, prostate and bowel were two of the most commonly diagnosed cancers worldwide and, with treatments emerging and evolving, survival rates for the majority of tumour types continue to increase [2]. Such increasing survival rates place huge importance on ensuring adequate levels of quality of life for patients, as life-extending cancer treatment regimens may result in increased symptom burden and decreased physical and emotional functioning.

Patient reported outcome measures (PROMs) are emerging as an important component for patient management in the cancer setting. PROMs are standardised and validated self-complete instruments which broadly provide patient perspective on domains relating to quality of life, symptom management, patient functioning and patient satisfaction with care or perceptions of care [3, 4]. Empirical evidence supports the use of PROMs in the clinical setting to identify patient concerns, enhance patient-clinician communication and improve patient satisfaction with care in the clinical setting [5, 6]. The widespread use of PROMs routinely is, however, limited, with the majority of use occurring within randomised control trials (RCTs) where PROMs are used to monitor health status and quality of life before, during and after experimental treatments. Additionally, PROMs are used in this setting to assess whether the survival benefits of a specific treatment may outweigh any potential side effects or for choosing between treatment options which offer similar survival benefit [7–9].

The use of PROMs in routine clinical practice is limited as healthcare services are challenged to sustain high care quality, whilst also facing increased demand and financial shortfalls [10]. Moreover, there exists a lack of established standard on what PROMs should be utilised in which setting and how benefit should be measured [11]. Indeed, despite the well-known benefits of PROMs in terms of quality of life, less is understood about the potential oncological benefits of utilising PROMs routinely in the clinical setting. Emerging evidence suggests a potential role for PROMs as independent prognostic tools which, when used alongside clinicopathological information, may provide clinicians with a more valid and comprehensive understanding of patient disease [12, 13]. A deeper understanding of this potentially prognostic function is imperative in order to develop a rationale for the widespread implementation of routine collection of PROMs within the clinical setting.

We therefore sought to systematically review the literature to determine current understanding of the potential prognostic role of PROMs, with reference to tumours of the pelvic-abdominal cavity specifically. Studies were critically appraised to identify any measurable oncologic benefit and are described using a narrative presentation.

## Methods

### Search strategy and inclusion criteria

The research question, search strategy, and inclusion and exclusion criteria were developed prior to commencement of literature searching in April 2020. Relevant studies were identified by conducting searches of Medline (Pubmed) and Ovid Gateway (Embase and Ovid) using the listed search terms from inception until April 2020. A comprehensive set of search terms was compiled and is included as a supplement (Additional file 1: Appendix A). After searching, the list of returned articles was further filtered to include only articles published in the English language and studies referring to humans alone. Reference lists of included articles were also checked for additional relevant literature.

The full inclusion and exclusion criteria are presented in Table 1. The following inclusion criteria was utilised: a randomised control trial, an observational study or an original article, written in the English language, and investigating the oncological outcomes of PROMs in patients with urological, gynaecological, colorectal, pancreatic, gastric or hepatic tumour types. Commentaries, author’s replies, reviews, supplements, editorials and systematic reviews were excluded. Studies that included patients with cancers other than pelvic abdominal tumours were included on the condition that the relevant pelvic abdominal cancer data could be isolated.

**Table 1:** Inclusion and exclusion criteria for screening

Inclusion criteria	Exclusion criteria
Peer reviewed paper	Non full text articles
Published at any time before April 2020	Non-English papers
	Systematic Reviews
*Quantitative analyses*	
Includes participants:	
Patients diagnosed with: Any urological cancer Any gynaecological cancer Any colorectal cancer Any gastric cancer Any hepatic cancer Any pancreatic cancer	Studies in which data pertaining to any of the included tumour types could not be isolated
Disease stage: any	
Treatment regimen: any	
Demographic: any	
*Quantitative studies*	
Design: Randomised control trial Prospective cohort Non-randomised control trial Cross sectional	
Variables examined: Prognostic potential of PROMs Specific QoL instruments with prognostic potential	

All duplicates were removed, and articles were reviewed by title, abstract and full text by the first author (CM). A second author (MVH) subsequently reproduced the results of the search strategy before independently undertaking screening of all articles included for full text review. In case of disagreement, a third independent reviewer (TGU) was consulted to confirm the final list of included studies. Management of the screening process occurred using Microsoft Excel.

### Quality assessment

Initially, the quality of each study was assessed by CM using quality assessment tools developed by the Joanna Briggs Institute (JBI) (http://joannabriggs.org/research/critical-appraisal-tools.html). The JBI have developed various tools for assessing the quality of quantitative studies that are appropriate for use in systematic reviews to appraise questions of aetiology and risk. The purpose of such appraisals is to broadly assess the methodological quality of a study and to determine the extent to which each study addresses the possibility of bias in its design, conduct and analysis. Owing to the varying design of the included studies, JBI critical appraisal checklists for cohort studies, randomised control trials and case series were utilised. The appraisals are included separately as Additional file 1: Appendix B1, B2 and B3. Following critical appraisal by CM, a second author (MVH) independently assessed the quality of each included study using the JBI critical appraisal tools. Each of the studies were subsequently discussed to identify any differences in opinion with consultation from a third author (TGU).

## Results

### Evidence synthesis

**Fig. 1:**
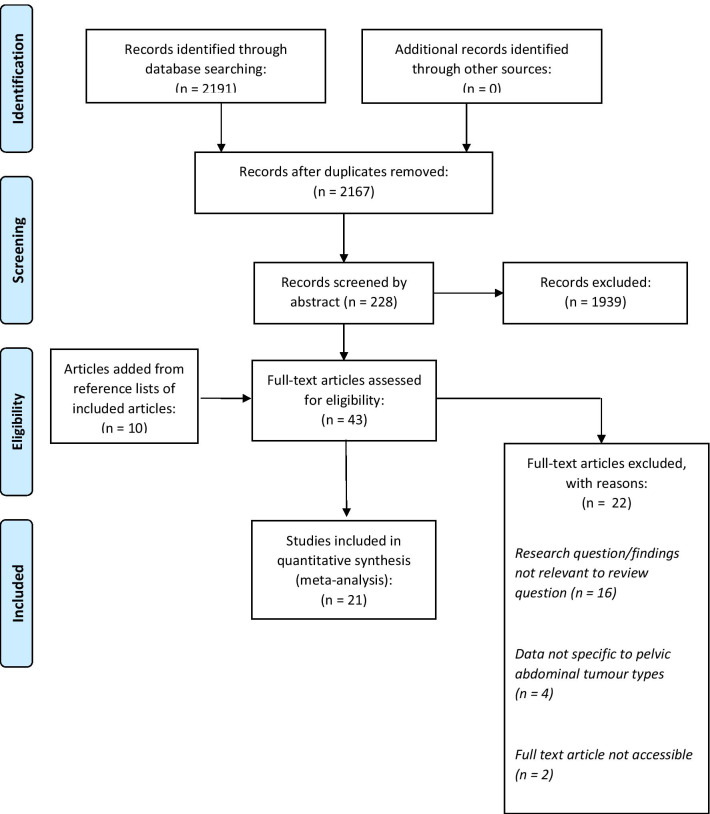
PRISMA flow diagram of screening process

As detailed in the PRISMA flow diagram (Fig. 1), the search strategy identified 2191 articles. Following the removal of 24 duplicates, and using the inclusion criteria outlined above, 2167 articles were screened by title. A further 228 records subsequently underwent abstract review before 43 were assessed based on the full text. Overall, 21 articles were deemed suitable for inclusion. Full details of the included studies are presented in Table 2.

**Table 2:** Descriptives of included studies

ID	References	Author, (date of publication)	Study population: no. of participants, age (mean (S.D.) and sex (n (%))	Tumour type, country of study	PROMs used	Study aims	Statistical analysis, covariates	Summary of findings
1	[22]	de Rooij et al. (2018)	N = 2457; Age: 68.1 (10.1); M: 1457 (59); F: 1000 (41)	Colorectal, Urological, Gynaecological The Netherlands	EORTC QLQ-C30, Hospital Anxiety and Depression Scale (HADS)	Identification of subgroups of cancer survivors with realistic, pessimistic or optimistic illness perceptions (IPs) relative to prognosis at time of questionnaire Assessment of HRQoL and survival associated with these subgroups	Multivariate Cox proportional Hazards regression models Clinical/demographic characteristics	Functioning and global QoL were higher and symptom burden lower in those with optimistic illness perceptions compared with realistic IPs (all *P* < 0.01) Functioning was lower and symptoms were higher in those with pessimistic IPs compared to those with realistic IP (all *P* < 0.01) All-cause mortality higher in survivors with pessimistic IPs compared with those with realistic IPs (HR 1.52, 95% CI (1.27–1.84)
2	[23]	Graham et al. (2018)	N = 68; Age: 64.15; M: 53 (77.9); F: 15 (22.1)	Renal United States	Edmonton Symptom Assessment System (ESAS)	Assessment of whether ESAS measured at baseline can provide prognostic information for patients receiving standard first-line sunitinib for metastatic renal cell carcinoma (mRCC)	Multivariate Cox proportional Hazards regression models, Known prognostic indicators of mRCC	In multivariate analysis, higher baseline symptom burden was associated with inferior overall survival (HR 1.21, 95% CI (1.01–1.44) for each 10-unit increase in the ESAS total score when controlling for the mskcc risk group and 1.240 (*P* = 0.019) for each 10-unit increase when controlling for the imdc risk group Baseline symptom burden, as measured by ESAS, provides modest degree of prognostic information independently of other widely used prognostic factors
3	[24]	Baekelandt et al. (2016)	N = 66; Age: 68 (34–83); M: 20 (30.3); F: 44 (69.7)	Pancreatic Norway	EORTC QLQ-C30, EORTC QLQ-PAN26, Edmonton Symptom Assessment System (ESAS)	Assess the prognostic significance of pretreatment HRQoL and symptom scores on survival in patients with resectable pancreatic ductal adenocarcinoma	Multivariate Cox proportional Hazards regression models Clinical/demographic characteristics	Six reported variables with *P* < 0.20 eligible for multivariate model. After stepwise backward selection only cognitive function remained in model Based on results of prognostic impact of PROs on survival, patients divided into 2 groups: those with high versus those with low cognitive function Hazard ratio for death (HR 3.5, 95% CI (1.7–7.3) higher in patients with low cognitive function compared to higher cognitive function
4	[25]	Moningi et al. (2015)	N = 110; Age: NI; M: NI; F:NI	Pancreatic United States	EORTC QLQ-PAN26	Examine associations between QoL as they relate to self-reported symptoms, clinical characteristics and performance status	Pearson's Chi-squared Test	Patients with lower performance status measured by both ECOG and KPS had worse QoL scores: pain, digestive symptoms, cachexia, ascites (*P* < 0.05). More aggressive symptom management may result in improved PFS and better outcomes for pancreatic patients
5	[14]	Bingener et al. (2015)	N = 431; Age: 69.0 (11.2); M: 219(50.8); F: 212 (49.2)	Colorectal United States	Symptom Distress Scale, 5-item Quality of Life Index	Investigate whether deficits in preoperative QoL scores are associated with surgical outcomes such as 30-day morbidity Determine whether change in QoL postoperatively associated with morbidity	Stepwise logistic and linear models Demographics	Changes from baseline to day 2 QoL indicators, including concentration (OR1.27 (1.00–1.61) *P* = 0.049), appearance (OR 1.38 (1.02–1.87) *P* = 0.037), breathing (OR 1.50 (1.02–2.21), *P* = 0.038) significantly associated with early complications Changes from baseline to day 14 in ‘activities’, ‘daily living’ and ‘total QLI’ were also associated with early complications. Using stepwise logistic model, the variables significantly associated with having any early complications (yes/no) were age, ASA III and change in ‘activity’ from baseline to day 14 Significant predictors for being readmitted to the hospital within 2 months were baseline pain distress severity and changes from baseline to day 2 in fatigue. Also associated with readmission were changes from baseline to postoperative day 14 in ‘daily living’ and outlook
6	[21]	Quinten et al. (2013)	N = 2603; Age: NI; M: NI, F: NI	Colorectal, Urological, Gynaecological, Pancreatic Belgium	EORTC QLQ-C30	Investigate the relative contribution of different HRQoL domains as prognostic for separate cancer types	Multivariate Cox proportional Hazards regression models Established clinical/socioeconomic prognostic indicators	Results demonstrated that, for each cancer site, at least 1 HRQOL domain provided prognostic information that was additive over and above clinical and sociodemographic variables. However, the HRQOL parameters of greatest prognostic value differed across the cancer groups; and the effect size of each HRQOL parameter, indicated by the HR, depended on the tumor site Physical functioning linked to survival in colorectal cancer (HR 0.93, 95% CI (0.96–0.99)) Nausea and vomiting significant assocation in colorectal and ovarian cancer; (HR 1.06, 95% CI (1.01–1.07) and (HR 1.16, 95% CI (1.07–1.25) respectively
7	[26]	Robinson et al. (2011)	N = 723; Age: NI; M: 0 (0), F: 723 (100)	Gynaecological Denmark	EORTC QLQ-C30	Hypothesised that diagnostic delay could be a indicator of poorer health care system performance, which may affect survival and patient satisfaction. Investigated associations between QoL, patient satisfaction and survival	Poisson regression modelling and multivariate Cox proportional Hazards models, Demographic/clinical factors	Association between QoL and survival differed between ovarian and endometrial cancer. In ovarian cancer significant association between increased fatigue and reduced survival (HR 1.84, 95% CI (1.09–3.10) In endometrial cancer significant association with survival remained after adjustment for physical functioning (HR 3.30, 95% CI (1.27–8.57), role functioning (HR 5.40, 95% CI (1.57–18.58), emotional functioning (HR 3.41, 95% CI (1.33–8.74), nausea (HR 2.7, 95% CI (1.13–6.45), appetite loss (HR 3.78, 95% CI (1.71–8.34)
8	[32]	Lehto et al. (2018)	N = 104; Age: 66.5 (51–82); M: 104 (100), F: 0 (0)	Prostate Finland	Ways of Coping Questionnaire, Anger Expression Scale, Life Experience Scale, Rotterdam Symptom Checklist, EORTC QLQ-C30, LENT-SOMA outcome measure	Investigated the baseline and early predictors of disease-free and overall survival times in prostate cancer patients of all ages and treated with external beam radiotherapy	Multivariate Cox proportional Hazard models, Demographic/clinical factors	Different QOL measures exhibited either a favorable or an unfavorable impact, i.e., an increased level of pain (HR 0.05; 95% CI 0.01–0.32) predicted longer survival, whereas prostate-area symptoms (HR 1.18; 95% CI 1.03–1.36), increased fatigue (HR 7.08; 95% CI 1.77–28.32), and reports of no or few physical symptoms (HR 9.90; 95% CI 1.48–66.30) were significant predictors of shorter survival time. However, when the overall quality-of-life index (total scale 1–7) was tested instead of the prostate-area symptom scale, it predicted a longer survival (HR 0.51, 95% CI 0.27–0.95, *P* = 0.033); when both scales were included, the effect of the overall QOL was weaker (HR 0.56; 95% CI 0.27–1.15, *P* = 0.113)
9	[27]	Gupta et al. (2015)	N = 917; Age: 63 (40.8–89.3); M: 917 (100), F: 0 (0)	Prostate United States	PS Questionnaire	Investigate whether self-rated health is a potential confounder of the relationship between patient satisfaction with service quality and survival in patients with prostate cancer undergoing treatment	Multivariate Cox proportional Hazard models, Demographic/clinical factors	Self-rated health found to be independent predictor of survival in multivariate analysis after controlling for patient satisfaction (HR 0.30, 95% CI (0.11–0.86). Finding coupled with observation that performance status and self-rated health correlated suggest that self-rated health is potential confounder in relationship between patient satisfaction and survival in prostate cancer
10	[28]	Jayadevappa et al. (2009)	N = 318; Age: 57.25 (4.75); M: 318 (100), F: 0 (0)	Prostate United States	SF-36, UCLA Prostate Cancer Index	Analysis of association between race/ethnicity, risk of biochemical recurrence and recovery pattern of patient reported outcomes and cost in younger men with newly diagnosed prostate cancer	Linear mixed effect models, Demographic/clinical factors	Ethnicity is not a predictor of generic and prostate-specific HRQoL after adjustment for demographic and clinical variables Low risk of biochemical recurrence was associated with better physical function, vitality, mental health general health, urinary function, bowel function, bowel bother and sexual bother
11	[34]	Lis et al. (2008)	N = 230; Age: 61.8 (40–87); M: 230 (100), F: 0 (0)	Prostate United States	The Ferrans and Powers Quality of Life Index	Aimed to determine whether patient satisfaction with HRQoL might predict length of survival in patients with prostate cancer undergoing routine oncology treatment	Multivariate Cox proportional Hazard models, Clinical factors	Patient satisfaction with health and physical subscale was significantly associated with survival (RR:0.82, 95% CI (0.67–0.98) Association independent of previous treatment history and Gleason score
12	[33]	Lis et al. (2006)	N = 55; Age: 56.2 (33–74); M: 31 (56.4), F: 24 (43.6)	Pancreatic United States	The Ferrans and Powers Quality of Life Index (QLI)	Determine whether patient satisfaction with QoL predicts length of survival in patients with pancreatic cancer undergoing care in a nonclinical trial setting	Multivariate Cox proportional Hazard models, Disease Stage	Four variables (health and physical subscale, family subscale, global QoL and stage at diagnosis) found to be significant upon univariate analysis. No QoL subscale found to be significant after adjustment for stage at disease although health and physical subscale marginally significant (RR: 0.94, 95% CI (0.89–1.00) (*P* = 0.053)
13	[15]	Bernhard et al. (2010)	N = 295, Age: 63 (26–83); M: 138(46.8), F: 157 (53.2)	Pancreatic Switzerland	Global linear-analogue self-assessment (LASA)	Investigated the prognostic value of QoL relative to CA-19, and the role of CA-19 in estimating palliation in patients with advanced pancreatic cancer receiving chemotherapy within RCT	Multivariate Cox proportional Hazard models and linear mixed effect models	At baseline, less pain and tiredness (i.e. less symptom burden) predicted better survival (HR 0.65, 95% CI (0.45–0.96)) and (HR 0.63, 95% CI (0.44–0.92)) respectively baseline CA 19–9 did not predict QOL or time on study treatment, besides a marginal effect on pain. Neither CA 19–9 nor QOL predicted tumour response to chemotherapy. Survival is influenced by different factors than response to chemotherapy, although response impacts on survival. Thus, CA 19–9 and QOL at baseline provide limited information for estimating palliation by chemotherapy
14	[16]	Gourgou-Bourgade et al. (2013)	N = 342; Age: 61 (25–76); M: 211(62.0), F: 131 (38.0)	Pancreatic France	EORTC QLQ-C30	To compare the quality of life (QoL) of patients receiving oxaliplatin, irinotecan, fluorouracil, and leucovorin (FOLFIRINOX) or gemcitabine as first-line chemotherapy and to assess whether pretreatment QoL predicts survival in patients with metastatic pancreatic cancer	Multivariate Cox proportional Hazard models, QoL domains/clinical factors	Performance status, constipation (HR 1.06, 95% CI (1.01–1.11)) and dyspnea (HR 1.06, 95% CI (1.00–1.14)) remained significant after backward and forward selection procedures
15	[29]	Braun et al. (2013)	N = 186, Age: 55.1 (24–85); M: 121(65.1), F: 65 (34.9)	Pancreatic United States	EORTC QLQ-C30	Investigate whether pretreatment QoL parameters as well as changes in QoL scores from baseline until 3 months after treatment could predict survival in patients with stage IV pancreatic cancer	Multivariate Cox proportional Hazard models, Demographic/clinical factors	Found that every 10-point increase in baseline global QoL score was associated with a 12% decreased risk of death (HR, 0.88; 95% CI, 0.81Y0.95; *P* = 0.001) Improvement in cognitive function at 3 months is an indicator of improved patients’ survival after adjusting for other covariates (HR 0.89, 95% CI (0.77–0.98))
16	[19]	Roychowdhury et al. (2003)	N = 364; Age: 63.5; M: 288(79.1), F: 76 (20.9)	Bladder United States	EORTC QLQ-C30	Analysis performed to determine the prognostic significance of HRQoL parameters on time-to-event end points in patients with locally advanced or metastatic bladder cancer who participated in a phase III randomized study comparing gemcitabine and cisplatin (GC) with methotrexate, vinblastine, doxorubicin, and cisplatin (MVAC)	Multivariate Cox proportional Hazard models, Demographic/clinical factors	Identified HRQoL parameters (physical functioning, anorexia, and fatigue) to be significant and independent prognostic factors for time-to-event end points Physical functioning: (HR:1.56, 95% CI (1.15–2.10)), Anorexia: (HR 1.84, 95% CI (1.36–2.49)), Fatigue: (HR 1.46, 95% CI (1.11–1.94)
17	[30]	Maisey et al. (2002)	N = 501; Age: NI; M: NI, F: NI	Colorectal United Kingdom	EORTC QLQ-C30	Examine the prognostic value of baseline QoL measurements in patients with locally advanced and metastatic colorectal cancer treated with systemic chemotherapy within the context of clinical trials	Multivariate Cox proportional Hazard models Clinical/demographic factors	Majority of the QoL domains remained significant independent predictors of overall survival in the final multivariate model: Physical functioning: (HR 1.35, 95% CI (1.07–1.69)) Role functioning: (HR 1.34, 95% CI (1.08–1.66)) Social fuctioning: (HR 1.43, 95% CI (1.13–1.81)) Emotional functioning: (HR 1.28, 95% CI (1.04–1.57))
18	[20]	Collette et al. (2004)	N = 391; Age: 70.7 (34.3–89.3); M: 391 (100), F: 0 (0)	Prostate Europe	EORTC QLQ-C30	Utilise HRQOL data from three RCTs to assess clinical and biochemical parameters to identify independent prognostic factors for overall survival	Stratified multivariate Cox proportional Hazard models	Symptom items of appetite loss and insomnia from the EORTC QLQ-C30 were retained as independent prognostic factors of overall survival: Appetite loss: (HR:1.47, 95% CI (1.16–1.86)) Insomnia: (HR:1.45, 95% CI (1.15–1.84)
19	[17]	Stucky et al. (2011)	N = 449; Age: 68.8 (11.25); M: 223 (50), F: 226 (50)	Colorectal United States	Symptom Distress Scale, 5-item Quality of Life Index	Evaluate the effect of baseline QOL on subsequent QOL and survival	Intention to treat analysis and stepwise logistic/linear regressions	Baseline outlook [hazard ratio (HR) = 0.58, 95% confidence interval (CI) 0.38–0.88, *P* = 0.01] and support (HR 2.85, 95% CI 1.52–5.35, *P* = 0.001) were significantly associated with overall survival
20	[18]	Chau et al. (2004)	N = 1080; Age: 62 (28–84); M: 842 (78), F: 238 (22)	Gastric United Kingdom	EORTC QLQ-C30	Identify baseline patient- or tumor-related prognostic factors and assess whether pretreatment QoL predicts survival in patients with locally advanced or metastatic oesophagus-gastric cancer	Multivariate Cox proportional Hazard models, Clinical/demographic factors	When pretreatment QoL data were tested against the baseline prognostic models, physical functioning (HR 0.76, 95% CI (0.60–0.97)) (*P* = .003), role functioning (HR 0.69, 95% CI (0.54–0.88) (*P* < .001), and global QoL (HR 0.57, 95% CI (0.45–0.72)) (*P* < .001) had significant prognostic impact
21	[31]	Coates et al. (1997), 47	N = 47; Age: 57.7; M: NI, F: NI	Gastric, Colorectal Australia, Germany, Canada	EORTC QLQ-C30	Evaluate the prognostic association of QL scores among patients with advanced malignancies in routine practice	Multivariate Cox proportional Hazard models, Clinical/demographic factors	Single-item QL scores for overall physical condition, overall quality of life, and the global and social functioning scales remained independently prognostic: Global QoL: (HR 0.99, 95% CI (0.98–0.99)) Social: (HR 0.93, 95% CI (0.98–0.99))

NI, no information

Of the 21 included studies, 7 were randomised control trials and 14 were observational in design. The articles were published between 1997 and 2018 and included sample sizes ranging from 47 patients to 2603. Seven of the studies were conducted on patients with pancreatic cancer; 6 on patients with a diagnosis of colorectal cancer; 5 on prostate cancer patients; 4 on urological cancers (including bladder and renal cancers); 3 on gynaecological cancers and 2 on gastric cancer patients. Although the studies were conducted worldwide, a large proportion (10/21) were published in the United States. The treatment setting of each included study also differed; 5 of the studies included patients undergoing chemotherapy regimens, 3 were conducted in the surgical setting, one study included survivors only and two studies included patients receiving targeted therapy or hormonal therapy/radiotherapy.

### Data presentation

Due to a lack of study homogeneity relating specifically to the patient reported outcome measurements utilised, quantitative synthesis was not viable and hence results are presented in a narrative style.

#### Randomised control trials

Of the seven RCTs included [14–20], two were interventions implemented in pancreatic cancer populations [15, 16], two were undertaken in a colorectal cancer setting [14, 17], one in bladder cancer [19], one in gastric cancer [18] and one in prostate cancer [20]. Two studies were based on the same RCT (Clinical Outcomes Surgical Therapy trial NCCTG 93-46-53), but investigated the prognostic significance of measuring baseline patient reported outcomes (PROs) in slightly different capacities [14, 17]. These two studies utilised results from a surgical RCT where the intervention compared open versus laparoscopic techniques in terms of post-operative complications and patient outcomes. Both studies used the Symptom Distress Scale and QOL Index recorded preoperatively to demonstrate the significant prognostic impact of such quality of life (QoL) measurements on the overall survival (OS) of colorectal cancer patients, and to demonstrate that these measures are more sensitive than clinician reported outcomes (CROs) in predicting mortality. Of note, longer term combined analysis of patients in both arms of the intervention identified lower patient baseline outlook as associated with decreased overall survival.

Four RCTs were secondary analyses of chemotherapy interventions whereby pancreatic, bladder and gastric cancer patients were randomised to receive varying regimens of cytotoxic therapy [15, 16, 18, 19]. Noteworthy observations from these studies included the prognostic significance of pain and fatigue as independent indicators for survival in pancreatic cancer, although these measures were found to be less prognostic than the carbohydrate antigen 19-9 (CA-19) [15]. In addition, this study surmised that QoL did not predict tumour response to chemotherapy.

A second RCT for pancreatic cancer patients utilised the EORTC QLQ-C30 questionnaire to identify no significant difference in QoL in treatment arms over time [16]. Subsequent multivariate analysis identified physical functioning, constipation and dyspnoea as significant prognostic factors in this patient cohort, with severely impaired physical functioning imparting the strongest negative effect on overall survival [16]. In the context of the bladder cancer RCT, Roychowdhury et al. also utilised the EORTC QLQ-C30 questionnaire to demonstrate physical functioning as a significant and independent prognosticator for time to event endpoints [19]. This RCT also identified a potential prognostic role for fatigue and anorexia in both treatment arms. Interestingly, in univariate analysis, higher role functioning was identified as a positive prognostic factor but, paradoxically, in the multivariate model longer overall survival was associated with lower role functioning.

In the setting of gastric cancer, three RCTs assessing fluorouracil-based combination chemotherapy were combined to investigate whether pretreatment QoL predicts survival in patients with locally advanced or metastatic disease [18]. In this study, and similarly to the other RCTs, better physical and role functioning predicted increased survival. Data was collated using the EORTC QLQ-C30 instrument and suggested the role of global QoL score as a strong prognostic factor.

In contrast to the other RCTs, a European hormonal therapy/radiotherapy intervention in prostate cancer identified that HRQoL factors, as measured by the EORTC QLQ-C30 instrument, did not accurately predict overall survival once clinical and biochemical factors were accounted for [20]. Despite baseline global health status being associated with overall survival in other tumour types, this relationship was not demonstrated in this study.

#### Cohort studies

Full details of the 11 included cohort studies are contained in Table 2. One was in the context of aggregated RCT data [21] and 10 were original cohorts [22–31]. The tumour breakdown of these patient cohorts were as follows: five analyses included urological cancer patients [21–23, 27, 28], four colorectal [21, 22, 30, 31], three gynaecological [21, 22, 26], three pancreatic [24, 25, 29] and one gastric cancer cohort [31].

An analysis by Jayadevappa et al. of 318 younger prostate cancer patients identified that low risk biochemical recurrence is mostly indicative of better generic and prostate specific HRQoL [28]. Similarly, an American analysis of 917 prostate cancer patients demonstrated that patient self-rated health is a potential confounder in the relationship between patient satisfaction and survival; thus suggesting that future studies investigating patient satisfaction should include collection of self-rated health modules [27]. In renal cancer, a study by Graham et al. observed that baseline use of the Edmonton Symptom Assessment System provides a modest degree of prognostic information about survival, independently of other widely used prognostic models [23]. These results were consistent with previously reported data examining the prognostic function of the FACT-KSI instrument.

In pancreatic cancer patient cohorts, analyses further suggested a role for the EORTC QLQ-C30 instrument to provide prognostic information for survival [29]. As reported above, baseline global health was an independent prognosticator and, interestingly, the study found that the probability of survival increased significantly if cognitive function improved within three months of treatment [29]. Of note, an analysis of 66 Norwegian pancreatic cancer patients also demonstrated that cognitive function, as measured by the Edmonton Symptom Scale, was an independent prognostic factor [24].

In the context of colorectal cancer, a large UK study of 501 patients identified that patients with high baseline global QoL have a 1-year survival that is almost double that of patients with a score below median value [30]. Multivariate analysis demonstrated that symptom and functioning measures, as recorded using the EORTC QLQ-C30, appeared to be a stronger predictor of overall survival compared to clinician measured performance status. A smaller analysis of 47 patients with advanced gastric or colorectal cancers identified that overall physical condition and global QoL was an independent prognosticator of overall survival [31]. Indeed, further preliminary analyses demonstrated an association between psychological response to cancer and survival.

A Danish analysis of a gynaecological patient cohort investigated patient quality of life and satisfaction as an alternate avenue for exploring the consequences of diagnostic delay [26]. This study identified that, in ovarian cancer patients specifically, pain was associated with reduced overall survival. In a subset of the cohort with endometrial cancer, a number of QoL domains including overall QoL, physical, emotional and role functioning, nausea and vomiting, pain, dyspnoea, and appetite loss were independently associated with survival [26].

#### Case series

Three studies recruited prostate and pancreatic cancer patients consecutively [32–34]. In the context of radiotherapy, overall survival and disease-free survival in prostate cancer patients with localised disease were predicted by socioeconomic status, psychological factors and patient self-reported QoL [32]. In this study, different QoL domains demonstrated favourable or unfavourable impact; patients with reports of few or no physical complaints predicted shorter survival whereas reported pain was prognostic for longer overall survival.

A case series of 55 pancreatic cancer patients identified a borderline significant association between baseline health and physical measures and survival after adjustment for disease stage at diagnosis [33]. Indeed, this study also suggested that patient satisfaction with QoL provides useful prognostic information.

## Discussion

This systematic review identified several domains of QoL as potential prognosticators for oncological outcomes in tumours of the pelvic abdominal cavity. Specifically, global QoL, physical and role functioning, and fatigue consistently emerged as independent prognostic factors for overall and disease-free survival across the included tumour types [14, 18, 19, 22, 26, 29–31, 33]. Other domains relating to pain, constipation, dyspnoea, anorexia and cognitive function also appeared to have a potential prognostic function, independently of the clinicopathological features of disease [14–16, 19, 24, 29].

Despite compelling published evidence observing the prognostic role of baseline QoL measurements, the causal relationship between QoL and overall survival remains enigmatic. It has previously been suggested that collection of self-report QoL measurements may indicate the underlying severity of disease more accurately than other crude clinical measurements such as tumour burden [35]. Indeed, previously published studies have observed the superior nature of QoL in assessing prognosis compared to tumour burden; Earlam et al. successfully utilised physical QoL score to predict the overall survival of colorectal patients with liver metastases receiving supportive care alone and identified that the extent of metastasis did not influence survival [36]. In addition, historical studies in lung cancer [37, 38] identified QoL as an independent prognostic factor for survival, but failed to identify a significant relationship between survival and number of metastatic sites or disease extent. It is hypothesised that tumour markers which accurately reflect tumour aggression may also impact patient QoL more significantly than tumour burden [30].

Equally, various studies have hypothesised that QoL may directly impact tumour behaviour and subsequent patient survival, although such evidence is limited and controversial. Various studies [39–41] suggested that improvements in QoL or patient mental and emotional wellbeing may influence survival, but were limited by small patient numbers. In comparison a larger study of more than 1000 head and neck cancer patients found no link between emotional wellbeing and survival in this patient group [42]. Overall, the potential underlying mechanistic action of QoL monitoring and impact on patient survival requires more investigation.

Despite the evident prognostic potential of PROMs, our overview of the literature suggests that most PROMs are collected in the context of scientific research rather than routinely in the cancer clinical setting. Although it is well-established that patient perspective is an integral component of high quality and patient-centred care [43], financial burden and logistical issues prevent many healthcare systems from adopting PROM collection [10], and there exists a lack of uniform approach for their implementation in cancer specifically [11]. The PRISMA study, which primarily surveyed clinicians from Europe or Africa, highlighted physician time constraints and patient factors as key barriers to PROM implementation within the palliative care setting [44]. Additionally, a lack of training and guidance for clinicians were identified in this study as factors preventing wide-spread roll-out, and Gibbons et al. identified difficulties relating to budget and available software tools as further barriers to successful implementation [45].

There also exists a lack of validated tumour specific outcome measurement tools in cancer. Within this review, the majority of studies utilised baseline measurements of the cancer generic EORTC QLQ-C30 instrument. Although a standard tool for measuring HRQoL, the instrument lacks sensitivity to subtle disease specific changes [46]. Importance therefore lies with the development and validation of disease specific instruments which can detect and quantify disease subtle changes and accurately inform treating clinicians and the patients [47].

It is also of note that this review was undertaken during the COVID-19 pandemic when fears around the longer-term burden of SARS-CoV-2 on cancer care were emerging. During the outbreak, outpatient cancer care underwent a perhaps perpetual paradigm shift towards remote telemedicine, which further highlighted the necessity of routine collection of PROMs to support patients and allow shared clinical decision-making.

### Limitations

Many of the analyses included large patient numbers and therefore the findings of this review can be assumed robust. There did, however, exist heterogeneity in the study design and methodology of the included studies which ultimately prohibited a collective meta-analysis of the reported data. Ten of the included studies were conducted using data collated in the United States where private health care is prevalent, and therefore the results of these studies may not be generalisable across other populations. Equally, most of the included analyses (13/21) covered pancreatic and prostate tumour types alone. Although the shorter and longer survival times associated with these cancers provide a comprehensive overview of quality of life across the full cancer journey, these cancers are analogous with a specific age range and the male sex. Therefore, the prognostic potential of QoL measures is less well understood in other tumour types of the pelvic abdominal cavity and may differ by sex and age. Further investigation into the prognostic potential of PROMs is warranted in such tumour types with limited existing evidence.

## Conclusions

Overall, the findings of this review suggest a role for the routine collection of baseline PROMs in tumours of the pelvic abdominal cavity to improve both patient quality of life and outcomes. Specifically, global QoL, physical and role functioning and fatigue consistently emerged as independent prognosticators indicative of survival across these tumour types.

## Supplementary Information

**Additional file 1:** Appendices A, B1, B2 and B3 detailing the search strategy and JBI quality assessments of each included study.

## Data Availability

Data sharing is not applicable to this article as no datasets were generated or analysed during the current study.
