# Supplementary material for: An assessment of the use of patient reported outcome measurements (PROMs) in cancers of the pelvic abdominal cavity: identifying oncologic benefit and an evidence-practice gap in routine clinical practice
Source: Health Qual Life Outcomes. 2021 Jan 15;19:20. doi: 10.1186/s12955-020-01648-x (PMC7810193; doi:10.1186/s12955-020-01648-x)
Supplement: Supplementary file 1 — Additional file 1: Appendices A, B1, B2 and B3 detailing the search strategy and JBI quality assessments of each included study. [file 12955_2020_1648_MOESM1_ESM.docx]

*Appendix A: Ovid MEDLINE search strategy*

1. (Kidney cancer* or renal cancer* or kidney neoplasm* or renal neoplasm* or prostate cancer* or prostatic cancer* or prostate neoplasm* or prostatic neoplasm*).mp. [mp=title, abstract, original title, name of substance word, subject heading word, floating sub-heading word, keyword heading word, organism supplementary concept word, protocol supplementary concept word, rare disease supplementary concept word, unique identifier, synonyms]

2. (Bladder cancer* or bladder neoplasm* or bladder tumor* or bladder tumour* or urinary bladder cancer* or urinary bladder neoplasm* or malignant tumor of urinary bladder*).mp. [mp=title, abstract, original title, name of substance word, subject heading word, floating sub-heading word, keyword heading word, organism supplementary concept word, protocol supplementary concept word, rare disease supplementary concept word, unique identifier, synonyms]

3. (Cervix cancer* or uterine cervical cancer* or cervical neoplasm* or cervix neoplasm* or uterine cervical neoplasm*).mp. [mp=title, abstract, original title, name of substance word, subject heading word, floating sub-heading word, keyword heading word, organism supplementary concept word, protocol supplementary concept word, rare disease supplementary concept word, unique identifier, synonyms]

4. (Vagina* cancer or vagina* neoplasm*).mp. [mp=title, abstract, original title, name of substance word, subject heading word, floating sub-heading word, keyword heading word, organism supplementary concept word, protocol supplementary concept word, rare disease supplementary concept word, unique identifier, synonyms]

5. (Ovary cancer* or ovarian cancer* or ovary neoplasm* or ovarian neoplasm*).mp. [mp=title, abstract, original title, name of substance word, subject heading word, floating sub-heading word, keyword heading word, organism supplementary concept word, protocol supplementary concept word, rare disease supplementary concept word, unique identifier, synonyms]

6. (Vulva* cancer* or vulva* neoplasm*).mp. [mp=title, abstract, original title, name of substance word, subject heading word, floating sub-heading word, keyword heading word, organism supplementary concept word, protocol supplementary concept word, rare disease supplementary concept word, unique identifier, synonyms]

7. (Endometr* cancer or endometr* neoplasm* or uterine neoplasm*).mp. [mp=title, abstract, original title, name of substance word, subject heading word, floating sub-heading word, keyword heading word, organism supplementary concept word, protocol supplementary concept word, rare disease supplementary concept word, unique identifier, synonyms]

8. (Fallopian tube cancer or fallopian tube neoplasm*).mp. [mp=title, abstract, original title, name of substance word, subject heading word, floating sub-heading word, keyword heading word, organism supplementary concept word, protocol supplementary concept word, rare disease supplementary concept word, unique identifier, synonyms]

9. (Hepatic cancer or liver cancer or hepatocellular cancer or hepatic neoplasm* or hepatic neoplasm* or hepatocellular neoplasm*).mp. [mp=title, abstract, original title, name of substance word, subject heading word, floating sub-heading word, keyword heading word, organism supplementary concept word, protocol supplementary concept word, rare disease supplementary concept word, unique identifier, synonyms]

10. (Gastric cancer* or stomach cancer* or gastric neoplasm* or stomach neoplasm*).mp. [mp=title, abstract, original title, name of substance word, subject heading word, floating sub-heading word, keyword heading word, organism supplementary concept word, protocol supplementary concept word, rare disease supplementary concept word, unique identifier, synonyms]

11. (Colon* cancer* or colon* neoplasm*).mp. [mp=title, abstract, original title, name of substance word, subject heading word, floating sub-heading word, keyword heading word, organism supplementary concept word, protocol supplementary concept word, rare disease supplementary concept word, unique identifier, synonyms]

12. (Pancreas cancer* or pancreatic cancer* or pancreas neoplasm* or pancreatic neoplasm*).mp. [mp=title, abstract, original title, name of substance word, subject heading word, floating sub-heading word, keyword heading word, organism supplementary concept word, protocol supplementary concept word, rare disease supplementary concept word, unique identifier, synonyms]

13. (Rect* cancer* or rectal tumour or rectal tumor or rect* neoplasm*).mp. [mp=title, abstract, original title, name of substance word, subject heading word, floating sub-heading word, keyword heading word, organism supplementary concept word, protocol supplementary concept word, rare disease supplementary concept word, unique identifier, synonyms]

14. (Anal cancer* or anal neoplasm* or anus cancer* or anus neoplasm*).mp. [mp=title, abstract, original title, name of substance word, subject heading word, floating sub-heading word, keyword heading word, organism supplementary concept word, protocol supplementary concept word, rare disease supplementary concept word, unique identifier, synonyms]

15. 1 or 2 or 3 or 4 or 5 or 6 or 7 or 8 or 9 or 10 or 11 or 12 or 13 or 14

16. (Patient reported outcome* or patient reported outcome measurements or PROM or patient satisfaction or PREM or PRO or outcome measure or outcome assessment or outcomes research or outcome study).mp. [mp=title, abstract, original title, name of substance word, subject heading word, floating sub-heading word, keyword heading word, organism supplementary concept word, protocol supplementary concept word, rare disease supplementary concept word, unique identifier, synonyms]

17. (Hrqol or health related quality of life or health-related quality of life or life quality or quality of life or QoL).mp. [mp=title, abstract, original title, name of substance word, subject heading word, floating sub-heading word, keyword heading word, organism supplementary concept word, protocol supplementary concept word, rare disease supplementary concept word, unique identifier, synonyms]

18. 16 or 17

19. (((Survey or survey) and questionnaire) or respond* or survey method* or assessment or report or relate* or apprais*).mp. [mp=title, abstract, original title, name of substance word, subject heading word, floating sub-heading word, keyword heading word, organism supplementary concept word, protocol supplementary concept word, rare disease supplementary concept word, unique identifier, synonyms]

20. exp Survival Rate/ or exp Disease-Free Survival/ or exp Survival/ or exp Progression-Free Survival/ or survival.mp. [mp=title, abstract, original title, name of substance word, subject heading word, floating sub-heading word, keyword heading word, organism supplementary concept word, protocol supplementary concept word, rare disease supplementary concept word, unique identifier, synonyms]

21. 17 or 20

22. 15 and 16 and 19 and 21

Appendix B1: JBI quality assessment of included randomised control trials

|  |  | S Gourgou-Bourgadeet al (2013) | J Bernhard et al (2010) | C.C.H Stucky et al (2011) | J Bingener et al (2015) | D.F. Roychowdhury et al (2003) | L Collette et al (2004) | I Chau et al (2004) |
| --- | --- | --- | --- | --- | --- | --- | --- | --- |
| 1 | Was true randomisation used for assignment of participants to treatment groups? | ✓ | ✓ | ✓ | ✓ | ✓ | ✓ | ✓ |
|  |  |  |  |  |  |  |  |  |
| 2 | Was allocation to treatment groups concealed? | x | x | x | x | x | U | U |
|  |  |  |  |  |  |  |  |  |
| 3 | Were treatment groups similar at baseline? | ✓ | ✓ | ✓ | ✓ | ✓ | ✓ | ✓ |
|  |  |  |  |  |  |  |  |  |
| 4 | Were participants blind to treatment assignment? | x | x | x | x | x | U | U |
|  |  |  |  |  |  |  |  |  |
| 5 | Were those delivering treatment blind to treatment assignment? | x | x | x | x | x | U | U |
|  |  |  |  |  |  |  |  |  |
| 6 | Were outcome assessors blind to treatment assignment? | U | U | x | U | x | U | x |
|  |  |  |  |  |  |  |  |  |
| 7 | Were treatment groups treated identically other than the intervention of interest? | ✓ | ✓ | ✓ | ✓ | ✓ | ✓ | ✓ |
|  |  |  |  |  |  |  |  |  |
| 8 | Was follow-up complete and, if not, were differences between groups in terms of follow-up adequately described and analysed? | ✓ | ✓ | ✓ | ✓ | ✓ | ✓ | ✓ |
|  |  |  |  |  |  |  |  |  |
| 9 | Were participants analysed in the groups to which they were randomised? | ✓ | x | ✓ | x | x | x | x |
|  |  |  |  |  |  |  |  |  |
| 10 | Were outcomes measured in the same way for treatment groups? | ✓ | ✓ | ✓ | ✓ | ✓ | ✓ | ✓ |
|  |  |  |  |  |  |  |  |  |
| 11 | Were outcomes measured in a reliable way? | ✓ | ✓ | ✓ | ✓ | ✓ | ✓ | ✓ |
|  |  |  |  |  |  |  |  |  |
| 12 | Was appropriate statistical analysis used? | ✓ | ✓ | ✓ | ✓ | ✓ | ✓ | ✓ |
|  |  |  |  |  |  |  |  |  |
| 13 | Was the trial design appropriate, and any deviations from the standard RCT design (individual randomisation, parallel groups) accounted for in the conduct and analysis of the trial? | ✓ | ✓ | ✓ | ✓ | ✓ | ✓ | ✓ |

Appendix B2: JBI quality assessment of included cohort studies

|  |  | J Graham et al (2018) | BH de Rooij et al (2018) | KM Robinson et al (2011) | D Gupta et al (2015) | R Jayadevappa et al (2009) | A Coates et al (1997) | D Braun et al (2012) | BM Baekelanet al (2016) | C Quinten et al (2013) | N Maisey et al (2002) | S Moningi et al (2015) |
| --- | --- | --- | --- | --- | --- | --- | --- | --- | --- | --- | --- | --- |
| 1 | Were the two groups similar and recruited from the same population? | ✓ | ✓ | ✓ | ✓ | ✓ | ✓ | ✓ | ✓ | ✓ | ✓ | ✓ |
|  |  |  |  |  |  |  |  |  |  |  |  |  |
| 2 | Were the exposures measured similarly to assign people to both exposed and unexposed groups? | ✓ | ✓ | ✓ | ✓ | ✓ | ✓ | ✓ | ✓ | ✓ | ✓ | ✓ |
|  |  |  |  |  |  |  |  |  |  |  |  |  |
| 3 | Was the exposure measured in a reliable and valid way? | ✓ | ✓ | ✓ | ✓ | ✓ | ✓ | ✓ | ✓ | ✓ | ✓ | ✓ |
|  |  |  |  |  |  |  |  |  |  |  |  |  |
| 4 | Were confounding factors identified? | ✓ | ✓ | ✓ | ✓ | ✓ | ✓ | ✓ | ✓ | ✓ | ✓ | ✓ |
|  |  |  |  |  |  |  |  |  |  |  |  |  |
| 5 | Were strategies to deal with confounding factors stated? | ✓ | ✓ | ✓ | ✓ | ✓ | ✓ | ✓ | ✓ | ✓ | ✓ | ✓ |
|  |  |  |  |  |  |  |  |  |  |  |  |  |
| 6 | Were the groups/participants free of the outcome at the start of the study (or at the moment of exposure)? | ✓ | ✓ | ✓ | ✓ | ✓ | ✓ | ✓ | ✓ | ✓ | ✓ | ✓ |
|  |  |  |  |  |  |  |  |  |  |  |  |  |
| 7 | Were the outcomes measured in a valid and reliable way? | ✓ | x | ✓ | ✓ | ✓ | ✓ | ✓ | ✓ | ✓ | ✓ | ✓ |
|  |  |  |  |  |  |  |  |  |  |  |  |  |
| 8 | Was the follow-up time reported and sufficient to be long enough for outcomes to occur? | ✓ | U | x | U | ✓ | ✓ | U | ✓ | ✓ | ✓ | U |
|  |  |  |  |  |  |  |  |  |  |  |  |  |
| 9 | Was follow up complete, and if not, were the reasons to loss to follow up described and explored? | U | U | U | U | ✓ | ✓ | U | U | ✓ | ✓ | U |
|  |  |  |  |  |  |  |  |  |  |  |  |  |
| 10 | Were strategies to address incomplete follow up utilised? | U | U | U | U | ✓ | ✓ | U | U | ✓ | U | U |
|  |  |  |  |  |  |  |  |  |  |  |  |  |
| 11 | Was appropriate statistical analysis used? | ✓ | ✓ | ✓ | ✓ | ✓ | ✓ | ✓ | ✓ | ✓ | ✓ | ✓ |

Appendix B3: JBI quality assessment of included case series

|  |  | C Lis et al (2008) | C Lis et al (2006 | U Lehto et al (2018) |
| --- | --- | --- | --- | --- |
| 1 | Were there clear criteria for inclusion in the case series? | ✓ | ✓ | ✓ |
|  |  |  |  |  |
| 2 | Was the condition measured in a standard, reliable way for all participants included in the case series? | ✓ | ✓ | ✓ |
|  |  |  |  |  |
| 3 | Were valid methods used for identification of the condition for all participants included in the case series? | ✓ | ✓ | ✓ |
|  |  |  |  |  |
| 4 | Did the case series have consecutive inclusion of participants? | ✓ | ✓ | ✓ |
|  |  |  |  |  |
| 5 | Did the case series have complete inclusion of participants? | ✓ | ✓ | x |
|  |  |  |  |  |
| 6 | Was there clear reporting of the demographics of the participants in the study? | ✓ | ✓ | x |
|  |  |  |  |  |
| 7 | Was there clear reporting of clinical information of the participants? | ✓ | ✓ | x |
|  |  |  |  |  |
| 8 | Were the outcomes or follow-up results of cases clearly reported? | ✓ | ✓ | ✓ |
|  |  |  |  |  |
| 9 | Was there clear reporting of the presenting site(s)/clinical(s) demographic information? | ✓ | ✓ | U |
|  |  |  |  |  |
| 10 | Was statistical analysis appropriate? | ✓ | ✓ | ✓ |
